# Genomic Tools in Pearl Millet Breeding for Drought Tolerance: Status and Prospects

**DOI:** 10.3389/fpls.2016.01724

**Published:** 2016-11-22

**Authors:** Desalegn D. Serba, Rattan S. Yadav

**Affiliations:** ^1^Agricultural Research Center-Hays, Kansas State University, HaysKS, USA; ^2^Institute of Biological, Environmental and Rural Sciences, Aberystwyth UniversityAberystwyth, UK

**Keywords:** pearl millet, *Pennisetum glaucum*, genomic tools, drought tolerance, GBS, QTL

## Abstract

Pearl millet [*Penisetum glaucum* (L) R. Br.] is a hardy cereal crop grown in the arid and semiarid tropics where other cereals are likely to fail to produce economic yields due to drought and heat stresses. Adaptive evolution, a form of natural selection shaped the crop to grow and yield satisfactorily with limited moisture supply or under periodic water deficits in the soil. Drought tolerance is a complex polygenic trait that various morphological and physiological responses are controlled by 100s of genes and significantly influenced by the environment. The development of genomic tools will have enormous potential to improve the efficiency and precision of conventional breeding. The apparent independent domestication events, highly outcrossing nature and traditional cultivation in stressful environments maintained tremendous amount of polymorphism in pearl millet. This high polymorphism of the crop has been revealed by genome mapping that in turn stimulated the mapping and tagging of genomic regions controlling important traits such as drought tolerance. Mapping of a major QTL for terminal drought tolerance in independent populations envisaged the prospect for the development of molecular breeding in pearl millet. To accelerate genetic gains for drought tolerance targeted novel approaches such as establishment of marker-trait associations, genomic selection tools, genome sequence and genotyping-by-sequencing are still limited. Development and application of high throughput genomic tools need to be intensified to improve the breeding efficiency of pearl millet to minimize the impact of climate change on its production.

## Introduction

The unfolding climate change; a rise in global average temperature caused mostly by increasing concentrations of greenhouse gasses in the atmosphere is expected to increasingly distress crop production in many parts of the world. An estimated 41% of the land area of the world constitutes arid and semiarid zones characterized by a very stochastic environment (Safriel et al., 2005), suboptimal for crop production due to drought. A crop species or genotype that is resilient to low rainfall amount and erratic distribution and elevated temperature would have a vital importance for sustainable food supply to the ever increasing world population. Developing a cultivar for such suboptimal environment through a carefully crafted breeding strategy is inevitable to sustainable food security in inhospitable climates.

Pearl millet [*Penisetum glaucum* (L) R. Br.] is a hardy cereal crop grown in the adverse climatic conditions of the arid and semiarid tropics where other cereals are likely to fail to produce economic yields. It is the staple food and fodder crop of millions of rural families in the hottest and driest environments of Africa and Indian subcontinent. As its cultivation is predominantly practiced on marginal lands under unirrigated condition, environmental stresses mainly drought and heat are the two most important production constraints that it faces.

A better understanding of the effects of drought on pearl millet and mechanisms of plant tolerance are vital for breeding new drought tolerant cultivars. However, complex mechanisms are involved in drought tolerance of crops and progress in developing drought tolerant varieties of pearl millet and other orphan crops have been slow (Bidinger et al., 1987; Howarth and Yadav, 2002; Yadav et al., 2002). Finding key functional components or machineries of drought tolerance would have utmost importance in overcoming this longstanding problem in breeding for drought tolerance. Application of molecular tools is proven to be quite important in dissecting complex quantitative traits such as drought tolerance into contributing traits, each controlled by QTL that follow Mendelian inheritance. With the advancements in next-generation sequencing (NGS) and molecular profiling technologies that is increasingly becoming more affordable and efficient to record DNA sequence variations over 1000s of genomic loci, identification and tagging of genes underling a trait in the genome have become possible.

In this review, the progress of development and application of genomic tools in breeding for drought tolerance in pearl millet are discussed. Some important morphological and physiological traits that can help in indirect selection, tools for molecular breeding approaches, and different approaches attempted are reviewed. The gaps in relation to breeding for drought tolerance, future prospects with respect to the application of genomic tools and potential platforms that can be used to accelerate the improvement of pearl millet for drought tolerance are examined.

## General Effects of Drought on Plant Growth and Development

Water is required by plants for transpiration, surface cooling and carbon assimilation and drought imposes an adverse effect on plant growth and development and the effect is manifold in nature. One of the key processes affected by water deficit in plants is photosynthesis due to decreased CO_2_ diffusion to the chloroplast (Pinheiro and Chaves, 2011) as stomata is closed to conserve available water in the plant system. Reduced photosynthesis can also be worsened by heat stress aggravated by water deficit (Crafts-Brandner and Salvucci, 2002). Decreased water in the plant system also damages chloroplast and affects photosynthetic outputs due to reduced light interception (Stone, 2001). Furthermore, the drought accompanying increased air temperature results in reduced reproduction due to heat damage on the pollen grain (Bita and Gerats, 2013), and increased sterility (Schoper et al., 1987; Jagadish et al., 2012).

Drought stress also prompt plants to accumulate metabolites such as proline that plays important role as protein precursors in plant metabolism and development. Proline accumulated in the plant cell serves as an excellent osmolyte, as a metal chelator, an anti-oxidative defense molecule, and a signaling molecule (Hayat et al., 2012). It is known to impart stress tolerance by maintaining cell turgor or osmotic balance, preventing electrolyte leakage by stabilizing membranes and proteins such as RUBISCO (Ribulose 1,5-bisphosphate carboxylase) (Hayat et al., 2012) and mitochondrial electron transport complex II (Hamilton and Heckathorn, 2001), and controlling reactive oxygen species (ROS) concentrations in plants (Paleg et al., 1984; Hare et al., 1998). Drought screening and molecular and biochemical evaluations conducted so far in pearl millet never considered the level of proline accumulation and its correlation with drought tolerance of genotypes. Some other metabolic changes associated with water deficit in the plant system include increased concentration of total soluble sugars, reducing sugars, sucrose, and amylase activity (Comino et al., 1997) which result in decreased starch grains. In general, reduction or inhibition of enzymatic activity, inhibition of cell expansion due to cell wall hardening to maintain turgor through lignification (Fan and Neumann, 2004), metabolic changes, ionic imbalance, disturbances in solute accumulation or a combination of two or more of these mechanisms are some of the effects of water shortfall in the plant system.

## Pearl Millet As A Drought Tolerant Cereal

Drought is the major constraint to pearl millet as it is grown in the drier semiarid and arid regions. However, adaptive evolution and natural selection made pearl millet relatively the most drought and heat tolerant among other cereals. Traditional landraces from drier regions are good sources for breeding drought tolerance (Kusaka et al., 2005; Yadav, 2010). Though, the yield potential of many of the landraces is lower as compared to improved cultivars, genes for drought tolerance in the landraces can be utilized to combine with high yield potential (Yadav et al., 2011).

Nevertheless, drought tolerance is a complex polygenic trait and influenced significantly by environments. The various morphological and physiological responses to drought are controlled by 100s of genes (Hu and Xiong, 2014). Plant stress responses involves adjustment of metabolism for physiological and morphological adaptation occurring as a result of dynamic and complex cross-talk between different regulatory networks (Saito and Matsuda, 2010). Several technical limitations prohibit the study of plant responses to environmental stress that appear to be complex and pose difficulty on the classical plant-breeding program. Apparently, the elucidation of the components of the complex mechanisms underlying drought tolerance in crops will accelerate breeding for drought tolerant cultivars.

The mechanism how plants endure the effect of drought on their growth and development takes various forms. The ability of plants to grow and yield satisfactorily with limited moisture supply or under periodic soil water deficits is termed as ‘drought resistance.’ It is principally the constitutive plant traits of maintaining high plant water status (dehydration avoidance) that enables plants to survive in water limited environments (Blum, 2005). This mechanism has virtuous relevance in terminal drought tolerance of pearl millet in which tolerant genotypes fill their grain and offset any drastic effect on their yield. Since phenotyping dehydration avoidance is difficult, no much study is devoted to understand the genetic bases of drought resistance in pearl millet and other related crops.

The other mechanism is the ability of plant to sense environmental factors and trigger development shift to mature before water stress becomes a serious limiting factor, which is termed as drought escape. The initiation of flowering in higher plants is a complex trait regulated by an integrated gene network that responds to the dynamics of both autonomous and environmental cues (Andres and Coupland, 2012). Flowering time is among the most common adaptive transitions (Zuellig et al., 2014) where ecologically relevant adaptive shifts taking place in plant development. Few recent studies shade light on the genetic factors underlying a shift in vegetative to reproductive stage and cycle length in pearl millet. A study on MADS-box gene family identified a polymorphism at a gene named *PgMADS11* was associated with flowering time variation in pearl millet (Mariac et al., 2011). The early shift from the vegetative to the reproductive stage warrant the survival of the species through successful completion of the life cycle before moisture deficit becomes worse, but with significant negative effect on the yield performance.

On the other hand, ability of a plant to withstand water deficit as measured by degree and duration of low plant water potential is termed as drought avoidance. This is characterized by water saving and water spending physiological and morphological mechanisms. Both root and shoot architecture are important in the drought avoidance mechanisms of plants. The water savers are plants which conserve water by closing of stomata when they sense water deficit in the soil. The water spenders have specialized root system to extract more water from deeper soil horizon or by adjusting their osmotic balance. Pearl millet root system is characterized by fast primary root development at early growth stage and quickly colonizing deeper soil horizon (Passot et al., 2016) that enables water acquisition from deeper root zone.

Plants that can recover from a dry period by producing new leaves or tillers from buds and able to survive drought by slowing total water loss are regarded as drought tolerant. In a highly drought prone environments pearl millet farmers specifically grow high tillering landraces (van Oosterom et al., 1996) that can grow secondary tiller when the main culm is affected by drought. These high tillering landraces usually have small panicle and small individual grain sizes (Aparna et al., 2014) to minimize the impairment of grain filling in drought conditions. As pearl millet grain yield is highly correlated with grain number (Bidinger and Raju, 2000), and an adaptive trait to the arid environment (DeWet et al., 1992), preference for high tillering landraces with small grain sizes that takes short period to fill the grain is a wise drought coping strategy helpful in combating extreme aridity.

## Morpho-Physiological Drought Adaptive Traits

Through evolution, the sessile plants evolved morpho-physiological traits that help them adapt to the dry environment. The morpho-physiological traits of importance in drought tolerance include both shoot and root characteristics naturally modified for efficient water absorption from the soil and conservation in the system. The morpho-physiological traits of drought tolerance effects relevant for yield are stomatal conductance, photosynthetic capacity, timing of phenological phases, starch availability during ovary/embryo development, stem reserve mobilization, stay green, single plant leaf area, rooting depth and density, cuticular resistance and surface roughness, osmotic adjustment, membrane composition, antioxidative defense, and accumulation of stress-related proteins (Cattivelli et al., 2008). More open stomata allows greater conductance and lower leaf temperature, a proxy for higher potential photosynthetic and transpiration rates. However, genotypes with higher stomatal conductance are relatively sensitive to drought stress as they lose water rapidly than those close their stomata and reduce their photosynthetic CO_2_ assimilation.

Increased mobilization of stem reserved assimilates to compensate for reduced current leaf photosynthesis in drought stress (Slafer et al., 2005), reduced leaf area through leaf rolling (Walter and Schurr, 2005), and leaf surface roughness that reduce water loss and induce greater light reflectance (Kerstiens, 1996) are some of the traits that have vital importance for drought stress tolerance.

Stay-green is a characteristic of some genotypes to extend the duration of active photosynthesis by delaying their leaves senescence for a longer period of time than other genotypes. Stay-green phenotype is induced by a complex signaling network that involves mutations suppressing ethylene, abscisic acid, brassinosteroid, and strigolactone signal transduction, or those activating cytokinin signaling otherwise regulating leaf senescence (Kusaba et al., 2013). The semi-dwarf pearl millet inbred lines developed in USA for hybrid breeding have stay-green characteristics. However, a detailed study of the genes involved or the region of the genome where such genes are residing is not conducted. A plant that maintains the integrity of its chlorophyll despite the soil water status during reproductive stage can continue photosynthesis and can fill the grain and maintain a reasonable grain yield performance in drought stress.

Root anatomy, architecture, depth, and density are also important traits in drought tolerance. Detailed characterization of pearl millet root system may have a significant contribution in pearl millet breeding for drought tolerance. Small fine root diameters and root length density are some of the root traits known to contributing to productivity under drought condition (Comas et al., 2013). Such type of genotypes are potential terminal drought tolerant for they can exploit the water available deeper in the soil profile when the upper root zone is dried. Neither the genes underlying such traits nor the genomic regions hosting the QTL for such root characteristics were investigated in pearl millet. Further, any genetic variation in the germplasm for such traits and its correlation with terminal drought tolerance was not studied yet.

## History of Genomic Tools Development and Application in Pearl Millet

**FIGURE 1:**
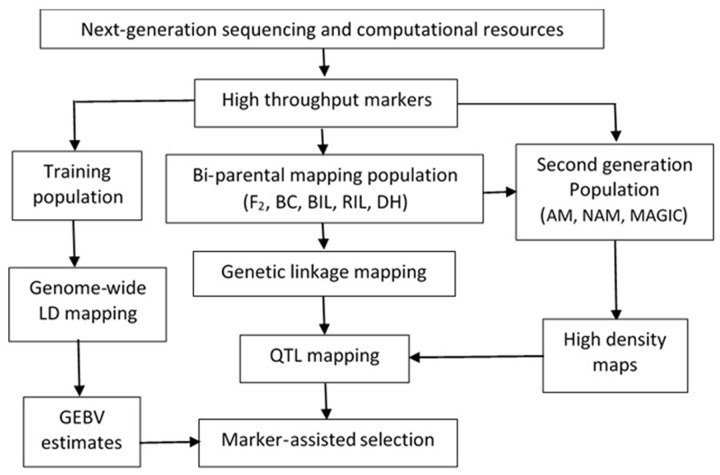
**Conceptual flow of molecular breeding using advanced genomic tools that can be applicable to pearl millet (F2, second filial generation; BC, backcross population; BILs, backcross inbred lines; RILs, recombinant inbred lines; DH, doubled haploid; AM, association mapping population; NAM, nested association mapping population; MAGIC, multi-parent advanced generation intercrossing population; GEBV, genetic and breeding value)**.

The application of genomic tools has become an essential component of breeding programs and proven to be useful for identifying novel genes for traits of agronomic importance and stress tolerance. Marker assisted-selection (MAS) and gene introgression into desirable genetic backgrounds have a great potential to facilitate crops improvement by cutting down lengthy phenotypic evaluation and selection. Alternative molecular plant breeding strategies have been developed for different crops based on the advancement in population genotyping (**Figure 1**).

The development of molecular markers for the improvement of pearl millet was conceived in 1991 by DFID-JIC-ICRISAT project (Gale et al., 2005). Consequently, the first genetic linkage map of pearl millet using restriction fragment-length polymorphisms (RFLPs) was constructed in Liu et al. (1994). Seven linkage groups corresponding to the haploid chromosome number of the species were formed.

The straight forward diploid genetics and high levels of polymorphism in pearl millet prompted several subsequent studies including mapping and tagging of genes controlling important traits in the pearl millet genome. The prominent work was the mapping of QTL to understand the genetic and physiological basis of terminal drought tolerance in pearl millet (Yadav et al., 2002). This has provided a more-targeted approach to improving the drought tolerance and yield of this crop in water-limited environments (Howarth and Yadav, 2002). A major QTL for terminal drought tolerance that explains more than 30% of grain yield variability in severe drought conditions was mapped on LG2 (linkage group two) using two independent mapping populations (Yadav et al., 2002; Bidinger et al., 2007).

Later, three major QTL for grain yield with low QTL × environment interactions were identified across a range of post-flowering moisture environments (Bidinger et al., 2007). The effect of this major QTL explaining more than up to 32% of the variations under drought was validated in two independent marker-assisted backcross programs (Serraj et al., 2005) in which 30% improvement in general combining ability for grain yield expected from this QTL under terminal drought stress was recovered in introgression lines (Yadav et al., 2011). Being predominantly climate resilient crops, millets may serve as valuable sources of novel genes for stress tolerance, which need to be identified and characterized. The close phylogenetic relationships between millets and other cereals could enable the introgression of novel alleles, genes or QTL identified in millets for better agronomic traits into other cereals toward ensuring food security under changing climate.

The major drought tolerance QTL identified by Yadav et al. (2002) has currently been found to be associated with reduced salt uptake (Sharma et al., 2014). A QTL for low transpiration rates that contributes to terminal drought tolerance through conserving soil moisture for grain filling by minimizing water lose during vegetative stage was co-mapped with the terminal drought tolerance QTL (Kholová et al., 2012). This co-mapping of the transpiration rate (a proxy trait to canopy conductance) and drought tolerance indicates that water conservation at a vegetative stage is important for the post-flowering stage performance of pearl millet. Reduced transpiration rate is an interplay of physiological traits such as reduced stomatal density and morphological traits of the canopy that plays important role in the rate of canopy conductance.

Development of PCR-based markers that can be used readily in diversity assessment and MAS programs have been conducted at different times. One way of developing SSRs was isolation from BACs through targeted use of 3′ end-anchored SSR primers and flanking sequences suppression PCR (Qi et al., 2001). This way of developing markers enables anchoring individual BACs to the genetic maps that in turn facilitate the construction of BAC contigs. EST database mining with the aim of developing PCR-based codominant markers found EST sequences containing di- and trinucleotide repeats at a density of 1.75 kb EST sequences (Senthilvel et al., 2008). Some of these EST-SSRs markers were found polymorphic among several set of parental lines used to develop mapping population for pearl millet genetic linkage mapping (Rajaram et al., 2013).

To integrate gene-based markers in the pearl millet genetic map, SNP markers developed from genes with known functions in plant adaptation to drought and associated abiotic stresses were used to identify candidate genes underlying major drought-tolerance QTL (Sehgal et al., 2012). Accordingly, genes coding for transferases, kinases, oxidases, and other proteins mostly associated with QTL of grain yield, flowering time and leaf rolling in drought stress conditions were co-mapped with the drought-tolerance QTL. This demonstrated the importance of functional markers in candidate gene identification and functional comparison of distantly related genomes in the study of drought tolerance and other traits. The candidate gene approach is preferred for QTL characterization than map-based cloning and insertional mutagenesis (Pflieger et al., 2001; Zhu and Zhao, 2007) as involvement of several genes with minor effect on the phenotype affect the efficiency of the later. In the identification of genes responsible for quantitative traits, candidate-gene approach using linkage maps based on functional gene markers has become essential (Sehgal et al., 2012). This approach of mapping functional gene markers in the QTL region was proven practical in putative candidate gene identification for drought tolerance in rice (Nguyen et al., 2004), barley (Tondelli et al., 2006), and durum wheat (Diab et al., 2008).

Development of high throughput markers has become quick and inexpensive through the development of NGS technology. Discovery of high throughput markers enabled genotyping of larger mapping population and construction of high and ultrahigh density maps in crops with no reference genome sequence available (Poland et al., 2012). As compared to other orphan crops, a sizable progress has been made for pearl millet in the development of molecular tools that are proven efficient in expediting the breeding of improved cultivars in the last two decades (Vadez et al., 2012). Development of molecular markers, EST libraries, BACs, and genome mapping were some of the areas in which progress has been observed. Nevertheless, to harness the wealth of genetic variability in the germplasm for improvement in yield, drought tolerance, and other desired traits much is remaining to be done (Vadez et al., 2012).

## Second-Generation Platforms For Molecular Breeding

The technological advances in NGS (Davey et al., 2011) also brought about a shift in linkage mapping population from the bi-parental to the second-generation multi-parent populations (Bohra, 2013) such as nested association mapping population (NAM) (Yu et al., 2008), multi-parent advanced generation intercross (MAGIC) population (Kover et al., 2009) to exploit multi-parent allelic diversity to a maximum advantage in fine mapping of QTL for desirable traits using an integrated mapping population. Use of second-generation populations with cutting-edge computing resources rendered a new opportunities in utilizing the advantages of both the linkage and association mapping for crop improvement (Bevan and Uauy, 2013).

The nested-association mapping utilizes the advantages of both linkage analysis and association mapping and eliminates disadvantages of both by taking into consideration recent and historical recombination events and facilitate high resolution mapping using an integrated multi-parent population (Yu et al., 2008). It has been utilized to identify functional markers or polymorphic sites that provide information about the genetic architecture underlying complex quantitative traits variability in many plant species (Andersen and Lübberstedt, 2003).

**FIGURE 2:**
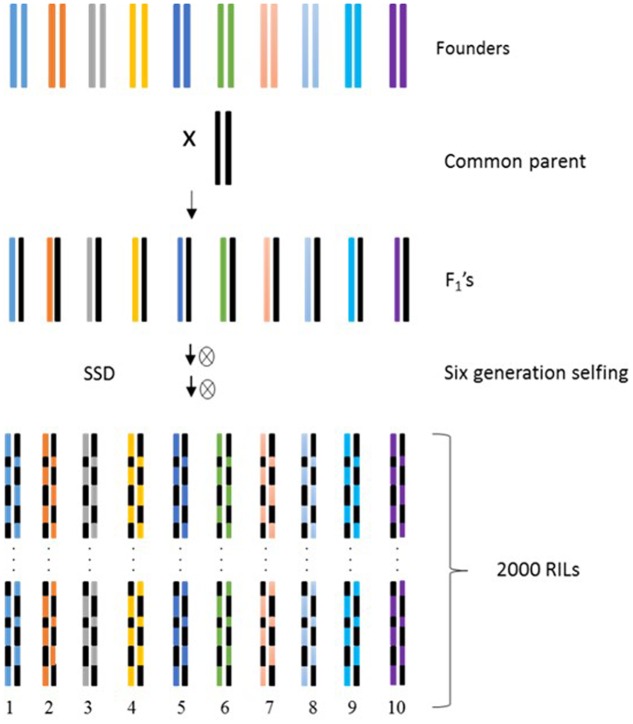
**Schematic diagram of pearl millet NAM population development.** The genome of each founder is color coded to show that the genome of RIL is a mosaic of founders and common parent genome segments (adapted from Yu et al., 2008). High-density genotyping of founders permits the linkage disequilibrium information captured in these diverse founders to be exploited for high-resolution mapping (SSD, single seed descent).

The NAM population should be developed in such a manner that it incorporates diverse parents crossed to a common parent (**Figure 2**). The information about the development and utilization of NAM in pearl millet to decipher allelic variations underlying agronomic and yield traits as well as tolerance to stresses including drought is lacking. As pearl millet exhibits high diversity, novel alleles for important traits can be captured through the utilization of such multi-parent population. As the cultivated pearl millet is classified into four races (typhoides, nigritarium, globosum, and leonis) (Brunken et al., 1977), second-generation mapping populations such as NAM, MAGIC and germplasm population needs to follow a comprehensive approach to incorporate such morphological variations and ethnographic isolates.

A germplasm based population [pearl millet inbred germplasm association panel (PMiGAP)] has been developed in pearl millet to capture wider genetic variations of traits (Sehgal et al., 2015). A structure analysis on this systematically developed association panel from ICRISAT global collection revealed six subpopulations corresponding predominantly to pedigree or similar agronomic traits (Sehgal et al., 2015). This result evidenced the importance of including different morphological forms in a molecular study for genome-wide marker discovery, QTL detection, or estimate of genomic based breeding value. Both within and between races assessment would likely help to discover novel allelic variants and extensive development of genomic tools for marker-assisted breeding.

**FIGURE 3:**
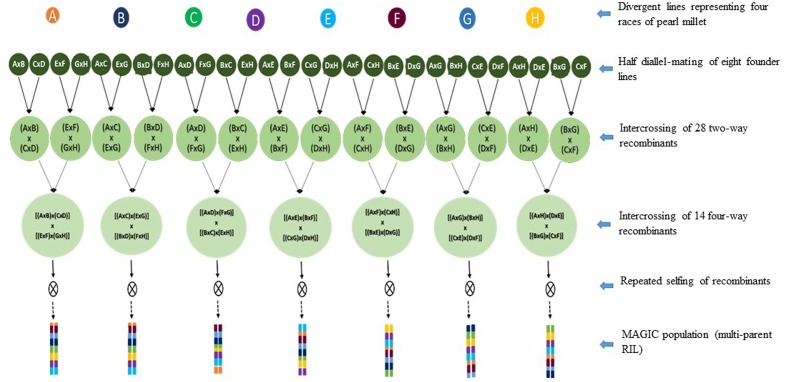
**Proposed crossing scheme for the development of multi-parent advanced generation inter-cross (MAGIC) population development for pearl millet.** The scheme involves half-diallel mating of eight molecularly diverse inbred germplasm, intercrossing of the recombinants, and repeated selfing to develop multi-parent recombinant inbred lines.

Multi-parent advanced generation intercross (MAGIC) population was proposed for genome-wide association mapping for the identification of major genes, QTL, and potentially novel loci associated with important traits (Kover et al., 2009; Bandillo et al., 2013). The analytical methodology involves reconstructing the genome of each line developed as a mosaic of the founders used to develop the population. It is expected to improve the genetic dissection of complex traits and precision of QTL mapping (Kover et al., 2009) for MAS and map-based cloning of candidate genes. The fixed multi-parent population in pearl millet will have advantages in assessing larger genetic variation in the germplasm than association analysis on the sample of naturally occurring haplotypes (Rakshit et al., 2012). Across species, a standard of 1000 fixed individuals developed from intercrossing of eight founders is advocated as the most appropriate for MAGIC (**Figure 3**). However, MAGIC has disadvantage mainly in resource utilization as it takes longer time and more resources to develop. The population is also likely to show extensive segregation for developmental traits, limiting their use in the analysis of complex traits.

The MAS uses molecular markers in linkage disequilibrium with major QTL that are tightly linked with the QTL and inherited together. The recent extension of marker-assisted selection is genomic selection (Meuwissen et al., 2001; Goddard and Hayes, 2007; Heffner et al., 2009) where genome-wide molecular markers are targeted in a large breeding population to improve quantitative traits. In genomic selection promising individuals are selected based on genomics estimated breeding values. It combines marker genotype data with phenotype from several environments and pedigree or kinship to estimate the breeding values. This method of selection based on genetic values predicted from molecular markers in the whole genome was anticipated to substantially speed up genetic gain than that achieved through selection based on major QTL (Meuwissen et al., 2001).

Genomic selection has been used for improvement of quantitative traits in several different crops with effective training population design. Being an orphan crop, pearl millet has not been evaluated yet for the selection strategy using breeding values. It was preferred to major QTL to accurately predict the genetic value of breeding lines using genome-wide high-throughput DNA markers information (Heffner et al., 2009). The major advantage of genomic selection lies in its potential to allow selection decisions during the off-season, thereby increasing genetic gain on an annual basis (Heffner et al., 2010). Development and utilization of genomic selection in pearl millet breeding can be applied in accurate prediction of the hybrid performance with ideal resource allocation. As has been surveyed in different crops (Zhao et al., 2015) it slightly improved prediction accuracies of hybrid performance when dominance effects were exploited.

## NGS Based Genotyping Platforms

Genotyping-by-sequencing (GBS) is the most widely used low-cost whole-genome marker profiling platform for a robust simultaneous marker discovery and genotyping (Elshire et al., 2011). This inexpensive highly multiplex protocol has been used in many crops for constructing libraries for NGS of genomes. A large number of high throughput single nucleotide polymorphisms (SNPs) markers were discovered and used to study within-species diversity and population structure, construct genome maps, and conduct genome-wide association studies (GWAS) (Metzker, 2010). Few works that have been conducted on pearl millet using this marker discovery and genotyping platform discovered important findings that encourage the application of the technology. High SNP polymorphism was observed between pairs of parental lines in gene based DNA sequences in pearl millet (Sehgal et al., 2012) indicating high allelic variation between the genotypes. Further, these markers are distributed across all the seven linkage groups that reflect the likely phenomenon of the distribution of the markers in the genome. Fine mapping of the DT-QTL on LG2 detected several markers mapped to the region that are associated with traits having prime importance in drought tolerance mainly delayed leaf senescence and leaf rolling under drought stress (Sehgal et al., 2012). GBS enabled construction of genetic map with high marker density and more uniform distribution than previously developed maps (Moumouni et al., 2015). As constructing a genetic map of a species is a key step in identifying and positioning a gene or QTL underlying a trait of interest (Pedraza-Garcia et al., 2010), marker density and uniform distribution play important role in the process of mapping and marker-trait association.

A recent molecular diversity and population structure analysis of 500 pearl millet accessions in two diversity panels (252 of global accessions from Africa, Asia, and the Americas; and 248 landraces from multiple agro-ecological zones in Senegal) using GBS discovered 83,875 SNPs that revealed the prevalence of high genetic diversity in accessions from Senegal as compared to the world collections (Hu et al., 2015). Rapid LD decay and lack of confounding population structure along agro-ecological zones in Senegal is predicted to simplify future pearl millet association mapping studies.

A genotyping method that allows an unbiased investigation of all exons or the complete protein-coding portion of the genome is exome sequencing. As only a small fraction of the genome is coding for protein (1–2%) (Warr et al., 2015), exome sequencing minimizes the sequencing space and associated cost. Either with *de novo* transcriptome assemblies for the development of array-based (Bi et al., 2012) or the solution-based whole-exome sequencing would have a practical application in pearl millet molecular breeding. The array-based is currently applicable to the pearl millet as no genome sequence available yet. One of the advantages of exome-sequencing commonly called exome-capture is more targeted development of SNP markers associated with variants of a gene controlling a phenotype of interest. Dealing with a small portion of the genome by excluding the repetitive parts also circumvents the complexity of data analysis and allow to handling larger numbers of samples than with other genotyping methods covering the whole-genome sequencing. It also enables to directly map mutations responsible for phenotypes of interest through direct sequencing of the exons.

## Pearl Millet Genome Sequence

The pearl millet genome sequence provides a genetic blueprint of the species and presumed to facilitate the development of genomic tools that would expedite the development of improved cultivars through the application of marker-assisted breeding. It also facilitate marker-trait association studies and identification of candidate genes for important quantitative traits such as yield and tolerance to stresses like drought. With the overall objective to sequence the pearl millet genome, ICRISAT spearheaded an informal International Pearl Millet Genome Sequencing Consortium (IPMGSC) that brought together several leading research organizations in genome sequencing, crop genomics, and pearl millet research^1^. The project of sequencing the pearl millet genome selected an important ancestral genotype of many seed parents of pearl millet hybrids in use for forage and grain in the USA as well as dual-purpose hybrids in India, Tift23D2B1.

The project successfully assembled large clean sequence data from whole genome shotgun sequencing (WGS) raw data (Varshney, 2014). It is expected that the first version of genome sequence will be published soon and apparently brings a paradigm shift to pearl millet molecular breeding by providing a foundation for accelerating gene mapping and characterization. The genome sequence needs to be complemented with transcriptome analysis to predict gene functions, their position in the genome, and expression patterns in different tissues, developmental stages and stress factors.

In pearl millet, there were several independent domestication events resulted in four different races (Brunken et al., 1977), enriching the genome sequence through resequencing of the populations used in the QTL mapping and in entirety of independently sequencing a genotype from other race thus becomes highly desirable. To conduct inclusive characterization of the genetic factors responsible for the seed morphological variations used as a basis for the classification, integrating the known races into the genome sequence would broaden the application of the resource.

## Future Prospects

The progress toward developing genomic resources for the application of NGS in pearl millet is promising but much awaits to implement genomics-assisted breeding to expedite the breeding for drought tolerance. With the development of such genomic tools, identification as well as development of improved genotypes is destined to become easier and faster. High density maps construction for fine mapping and candidate gene identification, integration with the genome sequence, and QTL detection needs to be strengthened. A shift from bi-parental to multi-parental and germplasm based association mapping platforms with the application of genomic tools can accelerate discovery of allelic variants that fast pace pearl millet improvement for drought tolerance.

Cost effective high throughput genotyping platforms are readily applicable in a diploid pearl millet genome. However, the respectable genome size should be taken into consideration in the process of selection for a genotyping method. The newly popularized exome-capture may reduce the extent of DNA sequence data handled and also leads to the identification of functional markers. Further, the advanced marker-assisted selection strategy that captures small-effect quantitative trait loci and breeding value needs to be given due emphasis for hybrid breeding. A release of the pending genome sequence would have paramount importance in the development and application of the aforementioned genomic tools for mapping and QTL detection. Integration of genome sequences with the consensus linkage map would increase the identification of genes underlying the QTL detected for drought tolerance.

## Author Contributions

DS conceived the idea and prepared the draft of the manuscript. RY edited the draft manuscript and contributed relevant information included in the review.

## Conflict of Interest Statement

The authors declare that the research was conducted in the absence of any commercial or financial relationships that could be construed as a potential conflict of interest.

## Footnotes

1 www.ceg.icrisat.org
